# Reconciling early-outbreak estimates of the basic reproductive number and its uncertainty: framework and applications to the novel coronavirus (SARS-CoV-2) outbreak

**DOI:** 10.1098/rsif.2020.0144

**Published:** 2020-07-22

**Authors:** Sang Woo Park, Benjamin M. Bolker, David Champredon, David J. D. Earn, Michael Li, Joshua S. Weitz, Bryan T. Grenfell, Jonathan Dushoff

**Affiliations:** 1 Department of Ecology and Evolutionary Biology, Princeton, NJ, USA; 2 Princeton School of Public and International Affairs, Princeton, NJ, USA; 3 Department of Biology, McMaster University, Hamilton, Ontario, Canada; 4 Department of Mathematics and Statistics, McMaster University, Hamilton, Ontario, Canada; 5 M. G. DeGroote Institute for Infectious Disease Research, McMaster University, Hamilton, Ontario, Canada; 6 Department of Pathology and Laboratory Medicine, University of Western Ontario, London, Ontario, Canada; 7 School of Biological Sciences, Georgia Institute of Technology, Atlanta, GA, USA; 8 School of Physics, Georgia Institute of Technology, Atlanta, GA, USA; 9 Division of International Epidemiology and Population Studies, Fogarty International Center, National Institutes of Health, Bethesda, MD, USA

**Keywords:** SARS-CoV-2, COVID-19, novel coronavirus, basic reproductive number, generation interval, Bayesian multilevel model

## Abstract

A novel coronavirus (SARS-CoV-2) emerged as a global threat in December 2019. As the epidemic progresses, disease modellers continue to focus on estimating the basic reproductive number $R_{0}$—the average number of secondary cases caused by a primary case in an otherwise susceptible population. The modelling approaches and resulting estimates of $R_{0}$ during the beginning of the outbreak vary widely, despite relying on similar data sources. Here, we present a statistical framework for comparing and combining different estimates of $R_{0}$ across a wide range of models by decomposing the basic reproductive number into three key quantities: the exponential growth rate, the mean generation interval and the generation-interval dispersion. We apply our framework to early estimates of $R_{0}$ for the SARS-CoV-2 outbreak, showing that many $R_{0}$ estimates are overly confident. Our results emphasize the importance of propagating uncertainties in all components of $R_{0}$, including the shape of the generation-interval distribution, in efforts to estimate $R_{0}$ at the outset of an epidemic.

## 1. Introduction

Since December 2019, a novel coronavirus (SARS-CoV-2) has been spreading globally [1]. Although the virus is likely to have originated from animal hosts [2], the ability of SARS-CoV-2 to directly transmit between humans, particularly without symptoms, has posed a greater threat for its spread [3]. As of 11 May 2020, more than 4 million cases of the coronavirus disease 2019 (COVID-19) have been confirmed internationally [4].

As SARS-CoV-2 began to spread in parts of China outside Hubei province, as well as in other countries, many analyses of the outbreak were published as pre-prints [5–10] and in peer-reviewed journals [11–14]. These analyses focused on estimating the basic reproductive number $R_{0}$—the average number of secondary cases generated by a primary case in a fully susceptible population [15,16]—in order to assess the pandemic potential of SARS-CoV-2. Rapid dissemination of these early analyses played an important role in shaping the response to the outbreak [17].

We commend these researchers for their timely contribution and those who made the data publicly available. However, the estimates of $R_{0}$ from different research groups (as well as the associated degrees of uncertainty) vary considerably even though most analyses rely on similar data—reports of confirmed cases from China, particularly from Wuhan City. Comparing a disparate set of estimates of $R_{0}$ can be difficult when the estimation methods and their underlying assumptions vary widely. In some cases, similar methods can give different estimates; in other cases, different methods can give similar estimates. Understanding the differences between $R_{0}$ estimates is critical to controlling an epidemic as $R_{0}$ provides information about the level of intervention required to prevent further transmission [15], and about the potential final size of the outbreak [15,18].

Here, we show that a wide range of approaches to estimating $R_{0}$ can be understood and compared in terms of estimates of three quantities: the exponential growth rate *r*, the mean generation interval $\bar{G}$ and the generation-interval dispersion *κ*. The generation interval, defined as the interval between the time when an individual becomes infected and the time when that individual infects another individual [19], characterizes the relationship between *r* and $R_{0}$ [20–23]; therefore, estimates of $R_{0}$ depend directly on their assumptions about the generation-interval distribution and the exponential growth rate. Early in an epidemic, information is scarce and there is uncertainty surrounding both case reports (affecting the estimates of the exponential growth rate) and contact tracing (affecting the estimates of the generation-interval distribution). Ignoring these uncertainties leads to overly confident conclusions.

To formalize the estimation of uncertainty at the onset of an outbreak, we present a statistical framework for averaging across estimates of the basic reproductive number $R_{0}$ from multiple studies. We apply the method to seven disparate models published online as pre-prints between 23 and 26 January 2020 that estimate $R_{0}$ for the SARS-CoV-2 outbreak in Wuhan City, China [5–10,24]. Previous studies have directly calculated the average of reported $R_{0}$ values [17,25] but such methods mask differences in underlying model assumptions and statistical methods. Instead, we model the estimate of $R_{0}$ (as well as the associated generation-interval parameters, $\bar{G}$, and *κ*) from each study with probability distributions that account for the uncertainty in the estimates; this allows us to re-estimate the corresponding distributions of the exponential growth rates *r*. We then use a Bayesian multi-level model to average the three key quantities (*r*, $\bar{G}$ and *κ*). The resulting pooled estimates (*μ*_*r*_, *μ*_*G*_ and $\mu_{\kappa }$) are used to calculate the pooled estimate of the basic reproductive number, $R_{\;\mathrm{pool}}$. Using pooled estimates allows us to average appropriately across the uncertainties present in modelling approaches and in their underlying assumptions. We use these pooled estimates to illustrate the importance of propagating different sources of error, particularly uncertainty in both the growth rate and the generation interval.

## 2. Methods

### 2.1. Description of the studies

We gathered information on estimates of $R_{0}$ for the SARS-CoV-2 outbreak in Wuhan City, China and their model assumptions from seven articles that were published online between 23 and 26 January 2020. Five studies [7–10,24] were uploaded to pre-print servers (bioRxiv, medRxiv and SSRN); one report was posted on the website of Imperial College London [6]; and one report was posted on nextstrain.org [5] (table 1).

**Table 1.** Summary of the models, analysed data, reported estimates of the basic reproductive number, and the assumptions about the generation-interval distributions. Model details, estimates of $R_{0}$ and their assumptions about the shape of the generation interval distributions were collected from seven studies. Generation-interval dispersion represent the squared coefficients of variation in generation intervals.

	model	data (study period)	data source	basic reproductive number $R_{0}$	mean generation interval $\bar{G}$ (d)	generation-interval dispersion *κ*	reference
study 1	deterministic branching process model	total number of cases in Wuhan City, China (through 18 Jan 2020)	estimated by Imai *et al.* [26]	1.5–3.5	10	1	Bedford *et al.* [5]
study 2	stochastic branching process model	total number of cases in Wuhan City, China (through 18 Jan 2020)	estimated by Imai *et al.* [26]	2.6 (1.5–3.5)^a^	8.4	not reported^b^	Imai *et al.* [6]
study 3	Poisson offspring distribution model	confirmed cases from China and other countries (29 Dec 2019–23 Jan 2020)	medical records and epidemiological investigations from Guangdong Province, China, and official websites of other regions in China	2.92 (95% CI: 2.28–3.67)	8.4	0.2	Liu *et al.* [7]
study 4	deterministic metapopulation susceptible–exposed–infected–recovered (SEIR) model	confirmed cases from China and other countries (1–21 Jan 2020)	not reported	3.8 (95% CI: 3.6–4.0)	7.6	0.5	Read *et al.* [9]
study 5	stochastic branching process model	total number of cases in Wuhan City, China (through 18 Jan 2020)	estimated by Imai *et al.* [26]	2.2 (90% CI: 1.4–3.8)	7–14	0.5	Riou & Althaus [24]
study 6	exponential growth model	confirmed cases from China (10–22 Jan 2020)	Wuhan Municipal Health Commission, China and National Health Commission of China	5.47 (95% CI: 4.16–7.10)^c^	7.6–8.4	0.2	Zhao *et al.* [10]
study 7	Incidence Decay and Exponential Adjustment (IDEA) model	reported cases from Wuhan City, China (1 Dec 2019–26 Jan 2020)	World Health Organization, National Health Commission of China, Wuhan Municipal Health Commission, and Huang *et al.* [27]	2.0–3.1	6–10	0	Majumder & Mandl [8]

^a^These intervals reflect $R_{0}$ values for best and worst scenarios. We treat these intervals as a 90% confidence/credible interval in our analysis.

^b^We assume *κ* = 0.5 in our analysis.

^c^The authors presented $R_{0}$ estimates under different assumptions regarding the reporting rate; we use their baseline scenario in our analysis to remain consistent with other studies, which do not account for changes in the reporting rate.

### 2.2. Model assumptions

Despite a wide range of models considered across the studies, all of them assume that the epidemic initially grows exponentially. The IDEA model (used in study 7) includes a discount parameter *d* that allows the model to deviate from exponential growth when *d* ≠ 0 [28], but study 7 estimates *d* = 0 across all parameters they consider. Even though some studies consider reported cases up to 26 January 2020—3 days after the travel restriction that took place on 23 January 2020 [29]—the exponential growth assumption can still describe the number of reported cases reasonably well; given the incubation period of around 5 days [30] as well as reporting delays of around 5 days [31], the majority of reported cases during the study periods are likely to have been infected prior to the travel ban.

When the epidemic is growing exponentially, the estimated basic reproductive number is determined by the exponential growth rate *r* and the intrinsic generation-interval distribution *g*(*τ*), which describes the infection time of secondary cases caused by a primary case in a fully susceptible population [32], via the Euler–Lotka equation [22]:

2.1 $$\frac{1}{R_{0}}=\int \exp(-r\tau )g(\tau )\;\text{d}\tau .$$

Therefore, it is sufficient to consider the estimates and assumptions about the exponential growth rates and the shapes of the generation-interval distributions to understand disparate estimates of the basic reproductive number. All model assumptions reduce to properties of the exponential growth rate *r* and the shape of the generation-interval distribution *g*(*τ*). For example, if a model relies on overly confident assumptions about the underlying observation (how new cases are reported) or process (how new cases are generated) model, the estimated confidence/credible intervals associated with the exponential growth rates or parameters of the generation-interval distributions (from each study) will necessarily be narrow.

As most studies do not report their estimates of the exponential growth rate, we first summarize model outcomes using reported (either estimated or assumed) values of the basic reproductive number $R_{0}$, mean generation interval $\bar{G}$ and generation-interval dispersion *κ*, represented by the squared coefficient of variation (table 1)—we re-estimate the corresponding exponential growth rates from these values later. Study 2 only reports their assumptions about the mean generation interval; for simplicity, we assume *κ* = 0.5 in our analysis. Study 6 presents $R_{0}$ estimates under 12 different scenarios regarding reporting rates (0-, 0.5-, one- or twofold increase in reporting rate) and the shapes of the generation-interval distributions based on previous coronavirus outbreaks (Middle East respiratory syndrome, MERS; severe acute respiratory syndrome, SARS; and their average)—we use their baseline scenario in our analysis to remain consistent with other studies, which do not account for changes in the reporting rate. While estimates of $R_{0}$ and the associated confidence intervals for study 6 in table 1 are based on $\bar{G}=8\;\text{d}$, we account for the uncertainty they consider for $\bar{G}$ in our formal analysis.

While most studies report confidence/credible intervals to quantify uncertainties associated with their estimates, some use different measures. In particular, study 2 reports a range of $R_{0}$ for the worst and best case scenarios, which correspond to the values of $R_{0}$ such that 95% and 5% of the simulated total number of cases by 18 January 2020 are greater than or equal to 4000, respectively; for simplicity, we treat these intervals as a 90% confidence/credible interval in our analysis. Uncertainty ranges reported by study 1 and study 7 are assumed to be uniform ranges.

Some of these studies have now been published in peer-reviewed journals [12,14] or have been updated with better uncertainty quantification [33]. As our primary focus is on the resolution of uncertainty in the available information during the earliest stages of an epidemic, rather than to provide more precise or accurate estimates of $R_{0}$, we focus strictly on estimates that were published between 23 and 26 January 2020.

### 2.3. Gamma approximation framework for linking *r* and $R_{0}$

Here, we use the gamma approximation framework to the generation-interval distribution [20,34–38] to (i) characterize the amount of uncertainty present in the exponential growth rates and the shape of the generation-interval distribution and (ii) assess the degree to which these uncertainties affect the estimate of $R_{0}$. The gamma distribution provides a reasonable approximation for generation-interval distributions of many diseases, including Ebola, measles and rabies [20]. Studies 1, 5, 6 and 7 also used a gamma distribution (including the special cases of Dirac delta and exponential distributions) to model the generation-interval distribution for SARS-CoV-2. Assuming that generation intervals follow a gamma distribution with mean generation interval $\bar{G}$ and generation-interval dispersion *κ*, represented by the squared coefficient of variation of a gamma distribution, we have [20]:

2.2 $$R_{0}={\left(1+\kappa r\bar{G}\right)}^{1/\kappa }.$$

This equation demonstrates that a generation-interval distribution that has a larger mean (higher $\bar{G}$) or is less variable (lower *κ*) gives a higher estimate of $R_{0}$ for the same value of *r* [22].

### 2.4. Re-estimation of the exponential growth rate

As most studies do not report their estimates of the exponential growth rate, we first re-estimate the exponential growth rate that corresponds to their model assumptions. Since the estimate of the basic reproductive number $R_{0}$ is determined by the exponential growth rate and the shape of generation-interval distributions, we can calculate the exponential growth rate from the basic reproductive number $R_{0}$, the mean generation interval $\bar{G}$ and the generation-interval dispersion *κ*. First, to account for uncertainties in these parameters, we model reported values of the basic reproductive number $R_{0}$, the mean generation interval $\bar{G}$ and the generation-interval dispersion *κ* with appropriate probability distributions. We use gamma distributions to model values reported with confidence/credible intervals (CI) and uniform distributions to model values reported with ranges; when confidence/credible intervals are reported, we parametrize the gamma distribution such that (i) its mean matches the estimated value and (ii) the probability that a random variable following the specified gamma distribution falls between the lower and upper confidence/credible limits is equal to the reported confidence/credible level. This probability is not necessarily based on equi-tailed quantiles. For example, study 3 estimated $R_{0}=2.92$ (95% CI: 2.28–3.67); we model this estimate as a gamma distribution with a mean of 2.92 and a shape parameter of 67, which has a 95% probability of containing a value between 2.28 and 3.67 (see table 2 for a complete description).

**Table 2.** Probability distributions for $R_{0}$, $\bar{G}$ and *κ*. We use these probability distributions to obtain a probability distribution for the exponential growth rate *r*. The gamma distribution is parametrized by its mean and shape. Constant values are fixed according to table 1.

	basic reproductive number $R_{0}$	mean generation interval $\bar{G}$ (d)	generation-interval dispersion *κ*
study 1	Uniform (1.5, 3.5)	10	1
study 2	Gamma (mean = 2.6, shape = 18)	8.4	0.5
study 3	Gamma (mean = 2.92, shape = 67)	8.4	0.2
study 4	Gamma (mean = 3.8, shape = 1400)	7.6	0.5
study 5	Gamma (mean = 2.2, shape = 12)	Uniform (7, 14)	0.5
study 6	Gamma (mean = 5.47, shape = 54)	Uniform (7.6, 8.4)^a^	0.2
study 7	$\exp{\left(r\bar{G}\right)}^{b}$	Uniform (6, 10)	0

^a^We do not account for this uncertainty during our re-estimation of the exponential growth rate *r* because the reported estimate of $R_{0}$ and its uncertainty assumes $\bar{G}=8$. We still account for this uncertainty in our pooled estimates (*μ*_*G*_).

^b^Instead of modeling $R_{0}$ with a probability distribution and re-estimating *r*, we use *r* = 0.114 days^−1^ (see text).

For each study *i*, we construct a family of parameter sets by drawing 10^5^ random samples from the corresponding probability distributions (table 2) that represent the estimates of ${\left(R_{0}\right)}_{i,m}$ and the assumed values of ${\bar{G}}_{i,m}$ and *κ*_*i*,*m*_ and calculate the exponential growth rate *r*_*i*,*m*_ by inverting equation (2.2):

2.3 $$r_{i,m}=\frac{{\left[{\left(R_{0}\right)}_{i,m}\right]}^{\kappa_{i,m}}-1}{\kappa_{i,m}{\bar{G}}_{i,m}},$$

where *m* = 1, …, 10^5^. This allows us to approximate the probability distributions of the exponential growth rates estimated by each study. Uncertainties in the probability distributions that we calculate for the estimated exponential growth rates reflect model assumptions, statistical methods, and also the quality of the data that each study relies on. This approach of re-estimating the exponential growth rate does not affect the uncertainty captured by our analysis because we are re-estimating the probability distribution of *r*_*i*_ that is consistent with the reported values of ${\left(R_{0}\right)}_{i}$, ${\bar{G}}_{i}$ and *κ*_*i*_; in other words, we still obtain the same degree of associated uncertainty in ${\left(R_{0}\right)}_{i}$ if we calculate it from *r*_*i*_, ${\bar{G}}_{i}$ and *κ*_*i*_.

For study 6, we fix $\bar{G}=8\;\text{d}$ and use the gamma distribution (table 2) that corresponds to $R_{0}=5.47$ (95% CI: 4.16–7.10) during the re-estimation step for *r* to remain consistent with the original study, which assumed $\bar{G}=8\;\text{d}$ for this particular estimate. We account for uncertainties in $\bar{G}$ for study 6 (table 1) in all other steps in order to properly incorporate parameter uncertainties in the estimate of $R_{0}$. Study 7 uses the IDEA model [28], through which the authors effectively fit an exponential curve to the number of confirmed cases without propagating any statistical uncertainty. Instead of modelling $R_{0}$ with a probability distribution and recalculating *r*, we use *r* = 0.114 d^−1^, which accounts for all uncertainty in the reported $R_{0}$ when combined with the considered range of $\bar{G}$ in the original article.

### 2.5. Pooled estimates

We construct pooled estimates for each parameter (*r*, $\bar{G}$ and *κ*) using a Bayesian multilevel modelling approach, which assumes that the parameter estimates across different studies are all drawn from the same gamma distributions:

2.4 $$\begin{array}{rl} \left(r_{1},\ldots ,r_{7}\right) & ∼\text{gamma}\;(\text{mean}=\mu_{r},\text{shape}=\mu_{r}^{2}/\sigma_{r}^{2}),\\ \left({\bar{G}}_{1},\ldots ,{\bar{G}}_{7}\right) & ∼\text{gamma}\;(\text{mean}=\mu_{G},\text{shape}=\mu_{G}^{2}/\sigma_{G}^{2})\\ \;\text{and}\;(\kappa_{1},\ldots ,\kappa_{7}) & ∼\text{gamma}\;(\text{mean}=\mu_{\kappa },\text{shape}=\mu_{\kappa }^{2}/\sigma_{\kappa }^{2}), \end{array}\}$$

where *μ*_*r*_, *μ*_*G*_, $\mu_{\kappa }$ represent the pooled estimates, and *σ*_*r*_, *σ*_*G*_ and $\sigma_{\kappa }$ represent between-study standard deviations. The pooled estimates, which are represented as probability distributions rather than point estimates, allow us to average across different modelling approaches while accounting for the uncertainties in their assumptions. Here, we do so by averaging across reported values, without explicitly re-fitting their models. We use a Markov chain Monte Carlo approach (cf. §2.7) and account for uncertainties associated with *r*_*i*_, ${\bar{G}}_{i}$ and *κ*_*i*_ (and correlations among them), by drawing a random set from the family of parameter sets $\left(r_{i,m},{\bar{G}}_{i,m},\kappa_{i,m}\right)$ for each study *i* at each Metropolis–Hastings step. Since the gamma distribution does not allow *κ* = 0 (this corresponds to a Dirac delta generation-interval distribution), we substitute *κ* = 0.02 for study 7. Although this approach nominally treats all studies equally, the overall pooled estimate will still be weighted by the certainty of the reported estimates (e.g. *r*_*i*_ will be sampled from a narrow distribution and therefore have stronger influence on *μ*_*r*_ if the reported confidence/credible interval on *r*_*i*_ is narrow).

Our approach does not account for non-independence between the parameter estimates made by different modellers. In this case, most estimates primarily depend on reported cases from China, particularly from Wuhan City. Differences among estimates are primarily driven by differences in estimation methods and underlying assumptions, rather than by epidemiological differences. The pooled estimates can become sharper (i.e. have narrower credible intervals) as we add more models even when the models or the data no longer add more information about the epidemic. Since SARS-CoV-2 spread primarily in Wuhan City, China, during this period, it is not possible to include independent sources of data from other countries. Thus, the pooled estimates should be interpreted with care.

### 2.6. Prior distributions

We use weakly informative priors on hyperparameters ($\mu_{r},\mu_{G},\mu_{\kappa },\sigma_{r},\sigma_{G},\sigma_{\kappa }$):

2.5 $$\begin{array}{rl} \mu_{r} & ∼\text{gamma}\;(\text{mean}=1/7\;{\text{d}}^{-1},\;\text{shape}=2)\\ \mu_{G} & ∼\text{gamma}\;(\text{mean}=7\;\text{d},\;\text{shape}=2)\\ \mu_{\kappa } & ∼\text{gamma}\;(\text{mean}=0.5,\;\text{shape}=2)\\ \; & (\sigma_{r},\sigma_{G},\sigma_{\kappa })∼\text{half-normal}\;(0,10). \end{array}\}$$

These priors are chosen such that their 95% quantile ranges are sufficiently wider than biologically realistic parameter ranges. Specifically, 95% quantile ranges for *μ*_*r*_, *μ*_*G*_ and $\mu_{\kappa }$ are $0.02-0.40\;{\text{d}}^{-1}$, 0.8–19.5 d and 0.1–1.4, respectively; 95% prior quantile range for $R_{0}$ then corresponds to 1.05–12.00. Parameters that are outside these ranges are biologically unrealistic for SARS-CoV-2 outbreaks. Therefore, we do not expect our results to be sensitive to these priors.

We follow recommendations outlined in Gelman *et al.* [39], parametrizing the top-level gamma distributions in terms of their means and standard deviations and imposing weakly informative prior distributions on between-study standard deviations, i.e. half-normal (0, 10). We initially used gamma priors with small shape parameters (<1) on between-study shape parameters (=*μ*^2^/*σ*^2^) but found this put too much prior probability on large between-study variances—a known problem [39]. Alternative choices of prior for the between-study shape parameters are also suboptimal. Imposing strong priors (e.g. half-*t* (*μ* = 0, *σ* = 1, *ν* = 4)) assumes *a priori* that between-study variance is large (and therefore does not pool different estimates sufficiently). Overly weak priors (e.g. half-Cauchy (0,5)) lead to inefficient sampling and poor convergence.

### 2.7. Markov chain Monte Carlo

We run four independent Markov chain Monte Carlo chains each consisting of 500 000 burnin steps and 500 000 sampling steps using the Metropolis–Hastings algorithm. Proposal distributions are modelled using independent normal distributions. Initial values and variances of the proposal distributions are chosen by trial-and-error to ensure a reasonable acceptance rate (around 10%) and convergence within 1 000 000 steps. Posterior samples are thinned to every 1000 steps to remove autocorrelations among posterior samples. Convergence is assessed by ensuring that the Gelman–Rubin statistic is below 1.01 [40] and the effective sample size is greater than 1000 for all hyperparameters ($\mu_{r},\mu_{G},\mu_{\kappa },\sigma_{r},\sigma_{G},\sigma_{\kappa }$); trace plots and marginal posterior distribution plots are presented in appendix A. Ninety-five per cent credible intervals (CI) are calculated by computing 2.5% and 97.5% quantiles from the marginal posterior distribution for each hyperparameter.

### 2.8. Comparing estimates of the basic reproductive number

In order to compare estimates of the basic reproductive number $R_{0}$ (and particularly their associated uncertainties) across different studies, we need a consistent measure of uncertainty. Instead of using reported uncertainty ranges from the original studies, we re-calculate the basic reproductive number from the parameter sets (*r*_*i*_, ${\bar{G}}_{i}$ and *κ*_*i*_) for each study using equation (2.2) and calculate the median and 95% equi-tailed quantile. We refer to these estimates as the base estimates. The distribution of the basic reproductive number for each study corresponds to the assumed distributions in table 2 for all studies except for study 6. The assumed distribution in study 6 in table 2 neglects uncertainty in the mean generation interval $\bar{G}$, whereas the base estimates account for this uncertainty. Furthermore, since the distributions in table 2 are constructed by matching the mean and the probabilities associated with the reported uncertainty ranges, the exact values of the base estimates and their 95% quantiles differ slightly from the reported values in table 1. We compare the base estimates with a pooled estimate of the basic reproductive number ($R_{\;\mathrm{pool}}$) based on the pooled estimates of underlying parameters (by substituting *μ*_*r*_, *μ*_*G*_, $\mu_{\kappa }$ in equation (2.2)).

### 2.9. Sensitivity analysis

In order to understand how uncertainties in each component (*r*_*i*_, ${\bar{G}}_{i}$ and *κ*_*i*_) affect the estimate of ${\left(R_{0}\right)}_{i}$ from each study *i*, we replace *r*_*i*_, ${\bar{G}}_{i}$ and *κ*_*i*_ with our pooled estimates (*μ*_*r*_, *μ*_*G*_ and $\mu_{\kappa }$, respectively) one at a time and recalculate the basic reproductive number $R_{0}$. We refer to the resulting estimates of $R_{0}$ as ‘substitute’ estimates. For example, the *r*-substitute estimate for study *i* is computed as:

2.6 $${\left(1+\kappa_{i}\mu_{r}{\bar{G}}_{i}\right)}^{1/\kappa_{i}},$$

where *κ*_*i*_ and ${\bar{G}}_{i}$ are taken from their corresponding parameter sets and *μ*_*r*_ is drawn from the posterior distribution. This procedure allows us to assess the sensitivity of the estimates of $R_{0}$ across appropriate ranges of uncertainties. We compare substitute estimates with the base estimates of $R_{0}$ (based on *r*_*i*_, ${\bar{G}}_{i}$ and *κ*_*i*_).

## 3. Results

**Figure 1.**
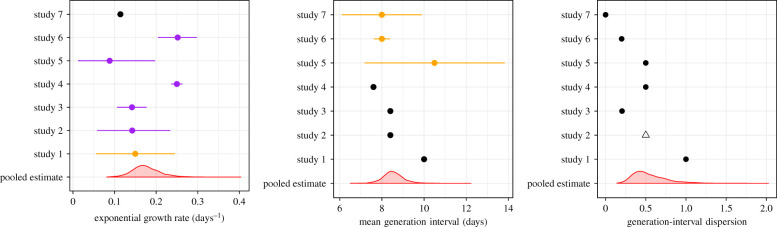
Comparisons of the reported parameter values with our pooled estimates. We inferred point estimates (black), uniform distributions (orange) or confidence/credible intervals (purple) for each parameter from each study, and combined them into pooled estimates using a Bayesian multilevel model (red). Points represent medians calculated from the parameter set $\left(r_{i},{\bar{G}}_{i},\kappa_{i}\right)$ for each study *i* (orange and purple). Error bars represent 95% equi-tailed quantiles calculated from the parameter set $\left(r_{i},{\bar{G}}_{i},\kappa_{i}\right)$ for each study *i*. Red density plots represent distributions of 2000 posterior samples. Open triangle: we assumed *κ* = 0.5 for study 2, which does not report generation-interval assumptions.

Figure 1 compares the estimated/assumed values of the exponential growth rate *r*, mean generation interval $\bar{G}$ and the generation-interval dispersion *κ* from different studies with the pooled estimates that we calculate from our multilevel model: *μ*_*r*_ = 0.17 d^−1^ (95% CI: 0.12–0.25 d^−1^), *μ*_*G*_ = 8.51 d (95% CI: 7.60–9.63 d) and $\mu_{\kappa }=0.50$ (95% CI: 0.26–1.10). Despite the large uncertainty associated with the underlying parameters, most studies consider narrower ranges of uncertainties in these parameters. No studies take into account how uncertainty in the generation-interval dispersion affects their estimates of $R_{0}$: all studies assumed fixed values for *κ*, ranging from 0 to 1. The estimates of the between-study standard deviations further suggest that there is a large variability in the underlying parameters among the seven studies, particularly in *r* and *κ*: *σ*_*r*_ = 0.07 d^−1^ (95% CI: 0.04–0.19 d^−1^), *σ*_*G*_ = 1.02 d (95% CI: 0.54–2.50 d) and $\sigma_{\kappa }=0.51$ (95% CI: 0.24–1.52). This variability is likely driven by the differences in modelling approaches and assumptions.

**Figure 2.**
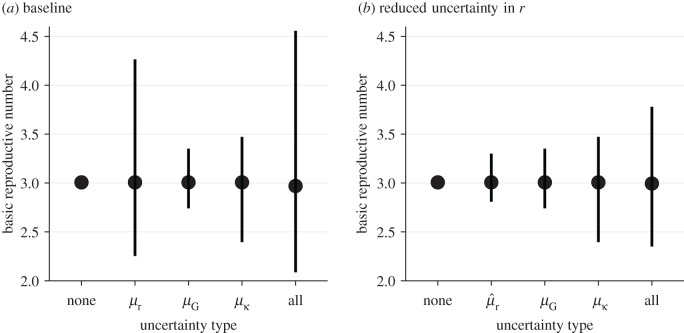
Effects of the exponential growth rate *r*, mean generation interval $\bar{G}$ and generation-interval dispersion *κ* on the estimates of the basic reproductive number $R_{0}$. We compare estimates of $R_{0}$ under nine scenarios that propagate different parameter uncertainties (*a*) based on our pooled estimates (*μ*_*r*_, *μ*_*G*_ and $\mu_{\kappa }$) and (*b*) assuming a fourfold reduction in uncertainty of our pooled estimate of the exponential growth rate (using ${\hat{\mu }}_{r}=(\mu_{r}+3\times \text{median}(\mu_{r}))/4$ instead of *μ*_*r*_). Each uncertainty type represents $R_{0}$ estimates based the posterior distributions of one of three parameters (*μ*_*r*_, *μ*_*G*_ and $\mu_{\kappa }$) while using median estimates of two other parameters. The ‘none’ type represents $R_{0}$ estimate based on the median estimates of *μ*_*r*_, *μ*_*G*_ and $\mu_{\kappa }$. The ‘all’ type represents $R_{0}$ estimates based on the joint posterior distributions of *μ*_*r*_, *μ*_*G*_ and $\mu_{\kappa }$ (also corresponds to $R_{\;\mathrm{pool}}$). Points represent the median estimates. Vertical error bars represent the 95% credible intervals.

Figure 2 shows how propagating uncertainty in underlying parameters affects estimates and CIs for $R_{0}$. For illustrative purposes, we use our pooled estimates, which may represent a reasonable proxy for the state of knowledge as of 23–26 January 2020 (figure 2*a*). Comparing the estimates that include only some sources of uncertainty to the pooled estimate ($R_{\;\mathrm{pool}}=3.0$; 95% CI: 2.1–4.6; see ‘all’ in figure 2), we see that propagating error from the growth rate (as done by all but one of the studies reviewed) is absolutely crucial: uncertainty in the pooled estimates for both middle bars (*μ*_*G*_ and $\mu_{\kappa }$), which lack growth-rate uncertainty, is overly narrow. In this case, propagating error from the mean generation interval has a negligible effect compared to propagating the uncertainty in *r*. Uncertainty in the generation-interval dispersion *κ* also has important effects (compare *μ*_*G*_ credible intervals with $\mu_{\kappa }$ credible intervals in figure 2*a*). However, our estimate of $R_{\;\mathrm{pool}}$ is relatively insensitive to our assumption of *κ* = 0.5 for study 2: assuming *κ* = 0.1 gives $R_{\;\mathrm{pool}}=3.0$ (95% CI: 2.2–4.7), whereas assuming *κ* = 0.9 gives $R_{\;\mathrm{pool}}=2.9$ (95% CI: 2.1–4.4).

We further explore how the effects of uncertainties in generation-interval distributions change when the estimate of the exponential growth rate is more certain. This hypothetical example reflects scenarios, in which increased data availability allows researchers to estimate *r* with more certainty. To simulate estimates of the exponential growth rate with narrower uncertainty, we use ${\hat{\mu }}_{r}=(\mu_{r}+3\times \text{median}(\mu_{r}))/4$ instead of *μ*_*r*_ (figure 2*b*); then ${\hat{\mu }}_{r}$ has the same median as *μ*_*r*_ but the associated 95% CI is four times narrower ($0.16-0.19\;{\text{d}}^{-1}$). As uncertainty associated with the exponential growth rate decreases, accounting for uncertainties in generation intervals becomes even more important. Propagating error only from the growth rate (${\hat{\mu }}_{r}$ in figure 2*b*) gives very narrow credible intervals in this case. Propagating errors from the mean generation interval (*μ*_*G*_ in figure 2*b*) or generation-interval dispersion ($\mu_{\kappa }$ in figure 2*b*) gives more realistic but still narrow credible intervals.

**Figure 3.**
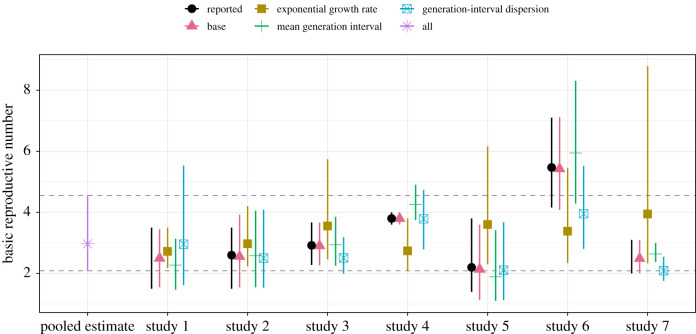
Sensitivity of the reported $R_{0}$ estimates with respect to our pooled estimates of the underlying parameters. We calculate substitute estimates by replacing the reported parameter values (growth rate *r*, mean generation interval $\bar{G}$ and generation-interval dispersion *κ*) with our corresponding pooled estimates (*μ*_*r*_, *μ*_*G*_ and $\mu_{\kappa }$) one at a time and recalculating $R_{0}$. The pooled estimate represents $R_{\;\mathrm{pool}}$, which is calculated from the joint posterior distribution of *μ*_*r*_, *μ*_*G*_ and $\mu_{\kappa }$; this corresponds to replacing all reported parameter values with our pooled estimates, which gives identical results across all studies. The reported estimates refer to estimates listed in table 1. Points represent the medians of the reported, base, substitute and pooled estimates. Vertical error bars represent the 95% credible intervals of our base, substitute and pooled estimates (based on 2000 posterior samples). Horizontal dashed lines represent the 95% credible intervals of our pooled estimate.

Finally, figure 3 compares the reported estimates (table 1) with the base estimates (based on *r*_*i*_, ${\bar{G}}_{i}$ and *κ*_*i*_ for each study *i*) as well as 21 substitute estimates (3 parameter substitutions × 7 studies). The base estimates, which are probability-based approximations of the reported estimates, are broadly consistent with the reported estimates. All but eight substitute estimates have wider credible intervals compared to their corresponding base estimates—the cases with more certain substitute estimates are the $\bar{G}$-substitute estimates for studies 1, 5 and 7, *r*-substitute estimates for studies 1 and 2 and *κ*-substitute estimates for studies 3, 6 and 7. Accounting for uncertainties in the estimate of *r* has the largest effect on the estimates of $R_{0}$ in most cases (figure 3). For example, the *r*-substitute estimate of $R_{0}$ for study 7 is $R_{0}=3.9$ (95% CI: 2.3–8.8), which is much wider than the uncertainty range reported by the authors (2.0–3.1). This is consistent with our earlier results (figure 2) that demonstrated the importance of accounting for uncertainty in the exponential growth rate *r*. In addition, the pooled estimate of the basic reproductive number ($R_{\;\mathrm{pool}}=3.0$; 95% CI: 2.1–4.6) has wider credible intervals than the base estimates for all studies except for study 6.

## 4. Discussion

Estimating the basic reproductive number $R_{0}$ is crucial for predicting the course of an outbreak and planning intervention strategies. However, comparing disparate estimates of $R_{0}$ can be difficult when they rely on different methods and assumptions. Here, we use a gamma approximation framework [20] to decompose $R_{0}$ estimates into three key quantities (*r*, $\bar{G}$ and *κ*) and apply a multilevel Bayesian framework to compare estimates of $R_{0}$ for the SARS-CoV-2 outbreak. Our results demonstrate the importance of accounting for uncertainties associated with the underlying generation-interval distributions, including uncertainties in the degree of dispersion in the generation intervals.

Our analysis shows that many early estimates of $R_{0}$ rely on overly confident assumptions. The neglect of uncertainties in the generation-interval dispersion is particularly important because it determines the shape of the *r*–$R_{0}$ relationship (figure 1): reducing *κ* from 1 (assuming exponentially distributed generation intervals) to 0 (assuming fixed generation intervals) changes the *r*–$R_{0}$ relationship from linear to exponential (see equation (2.2)). Assuming fixed parameter values here will lead to overly confident conclusions [41].

Omitting consideration of uncertainty in the generation-interval dispersion also explains the sensitivity of $R_{0}$ estimates to the exponential growth rate, particularly in study 7 (figure 3). Since study 7 assumes a fixed generation interval (*κ* = 0), they implicitly assume an exponential *r*–$R_{0}$ relationship, making their estimate too sensitive to *r*. Similarly, the credible intervals associated with the base estimates of studies 3 (*κ* = 0.2), 6 (*κ* = 0.2) and 7 (*κ* = 0) are wider than the credible intervals associated with their corresponding *κ*-substitute estimates, which rely on wider generation-interval distributions ($\mu_{\kappa }=0.50$; 95% CI: 0.26–1.10) and, therefore, are less sensitive to uncertainties in *r* and $\bar{G}$. One exception is study 1: this estimate of $R_{0}$ is most sensitive to generation-interval dispersion *κ*, because the study assumes an exponentially distributed generation interval (*κ* = 1). Estimates that rely on this assumption implicitly assume a linear *r*–$R_{0}$ relationship.

As most studies rely on overly confident assumptions, the credible intervals associated with the base estimates of $R_{0}$ should tend to be narrower than the credible intervals of the pooled estimate ($R_{\;\mathrm{pool}}=3.0$; 95% CI: 2.1–4.6). While the point estimate of $R_{\;\mathrm{pool}}$ is similar to other reported values from this date range, its credible interval is wider than the credible intervals of the base estimates of all but one study. This result does not mean that assumptions underlying the pooled estimate are too weak; rather, this credible interval more accurately reflects the level of uncertainties present in the information that was available when these models were fitted. In fact, because the pooled estimate does not account for overlap in data sources used by the models, it is more likely to be over-confident than under-confident. Because our median estimate averages over the various studies, particular studies have higher or lower median estimates. In particular, while the baseline example we used from study 6 may appear to be an outlier, the authors of this study also explore different scenarios involving changes in reporting rate over time, under which their estimates of $R_{0}$ are similar to other reported estimates.

Of the seven studies that we review, at least one of them directly fit their models to the cumulative number of confirmed cases. This approach is appealing because of its simplicity and apparent robustness, but fitting a model to cumulative incidence neglects autocorrelation between successive counts of cumulative cases. As a result, this approach both biases parameter estimates and gives overly narrow confidence/credible intervals [42,43]. Narrow uncertainties in the estimates of the exponential growth rate are probably driven by this approach.

Many sources of noise affect real-world incidence data, including both dynamical, or ‘process’, noise (randomness that directly or indirectly affects the actual number of cases occurring); and observation noise (randomness underlying how many of these cases are reported). Disease modellers face the choice of incorporating one or both of these in their data-fitting and modelling steps. Neglecting one or the other is not always a serious problem, particularly if the goal is inferring parameters rather than directly making forecasts [43]. Modellers should, however, be aware that oversimplifying the error model can give overly narrow confidence/credible intervals [42,44].

Our simple framework neglects some other important phenomena. Examples that seem relevant to this outbreak include: changing reporting rates; reporting delays (including the effects of weekends and holidays); and changing generation intervals. For emerging pathogens such as SARS-CoV-2, there may be an early period of time when the reporting rate is very low due to limited awareness or diagnostic resources; for example, Zhao *et al.* [10] (study 6) demonstrated that estimates of $R_{0}$ can change from 5.47 (95% CI: 4.16–7.10) to 3.30 (95% CI: 2.73–3.96) when they assume twofold changes in the reporting rate between 17 January, when the official diagnostic guidelines were released [45], and 20 January. Delays between key epidemiological timings (e.g. infection, symptom onset and detection) can also shift the shape of an observed epidemic curve and, therefore, affect parameter estimates as well as predictions of the course of an outbreak [46]. Even though a time-invariant delay between infection and detection may not affect the estimate of the growth rate, it can still affect the associated credible intervals. Other factors related to reporting—including changes in case definition, saturation in diagnostic test capacity, transparency of data, and representativeness of samples—will also affect estimation and inference. Finally, generation intervals can become shorter throughout an epidemic, as intervention strategies such as isolation of detected cases can reduce the infectious period [47]; since we are primarily focusing on the outbreak in Wuhan City before confinement, generation intervals are unlikely to change significantly. All of these factors, including fitting to cumulative curves or ignoring process error, affect the estimation of the exponential growth rate (as well as the associated uncertainties), which in turn affects the estimation of the basic reproductive number. Emergence of a new strain with different transmissibility could also affect disease dynamics, and complicate inference; this study does not address this possibility.

Here, we focus on the estimates of $R_{0}$ that are published within a very short time frame (23–26 January 2020). Since these estimates were published as pre-prints, rather than in peer-reviewed journals, the quality of the analyses as well as the resulting estimates were not necessarily finalized. For example, study 4 initially estimated $R_{0}=3.8$ (95% CI: 3.6–4.0; Read *et al.* [9]) but revised their estimate on 28 January 2020 to $R_{0}=3.11$ (95% CI: 2.39–4.13; Read *et al.* [33]); we do not include their revised estimates in our analysis in order to focus on information available at the very beginning of the outbreak. Some studies also lack detailed description of their methods, data, and/or assumptions. The variation in quality of these analyses adds further uncertainty to their results that is not captured by their uncertainty quantification (e.g. reported confidence/credible intervals) or by our analysis.

During early phases of an outbreak, it is reasonable to assume that the epidemic grows exponentially [15]. However, as the number of susceptible individuals decreases or behaviour changes in response to perception of the epidemic, the growth rate will decrease: estimates of *r* used for $R_{0}$ should account for the possibility that *r* is decreasing through time. Although our analysis applies strictly to the earliest stages of an epidemic, we expect certain lessons to hold more generally: confidence/credible intervals must combine as many sources of uncertainty as possible. In fact, as epidemics progress and more data become available, it is likely that inferences about exponential growth rate (and other epidemiological parameters) will generally become more precise; thus the risk of over-confidence (when uncertainty about the generation-interval distribution is neglected) will become greater. Incorporating estimates of the dynamics of susceptibility (e.g. using properly calibrated serological studies [48]) is also important for characterizing transmission as the outbreak progresses.

We strongly emphasize the value of attention to accurate characterization of the transmission chains via both contact tracing and improved statistical frameworks for inferring generation-interval distributions from such data [49]. A combined effort between public-health workers and modellers in this direction is crucial both for predicting the course of an epidemic and for controlling it. We also emphasize the value of transparency from modellers. Model estimates during an outbreak, even in pre-prints, should include code links and complete explanations. Methods based on open-source tools allow for maximal reproducibility [50].

Despite our focus on estimating $R_{0}$ at the onset of an outbreak, many of the issues persist now. For example, Flaxman *et al.* [51] recently estimated the basic reproductive number for SARS-CoV-2 outbreaks in 11 European countries to be around 3.8 (2.4-5.6), on average. While these estimates appear to be broadly consistent with earlier estimates from China, comparing the exponential growth rate and the underlying generation-interval distributions suggest otherwise. The later paper assumes a shorter mean generation interval ($\bar{G}=6.5\;\text{d}$) but similar generation-interval dispersion (*κ* = 0.38); based on these values, the exponential growth rate has to be considerably higher (*r* = 0.27 d^−1^) to obtain $R_{0}=3.8$ than the exponential growth rate observed in China (*μ*_*r*_ = 0.17 d^−1^; 95% CI: $0.12-0.25\;{\text{d}}^{-1}$). Naively comparing estimates of the basic reproductive number without accounting for differences in underlying assumptions can lead to over-interpretation of apparent differences in the estimates.

We have provided a basis for comparing exponential-growth based estimates of $R_{0}$ and its associated uncertainty in terms of three components: the exponential growth rate, mean generation interval and generation interval dispersion. We hope this framework will help researchers understand and reconcile disparate estimates of disease transmission early in an epidemic.

## Figures and Tables

**Figure 4.**
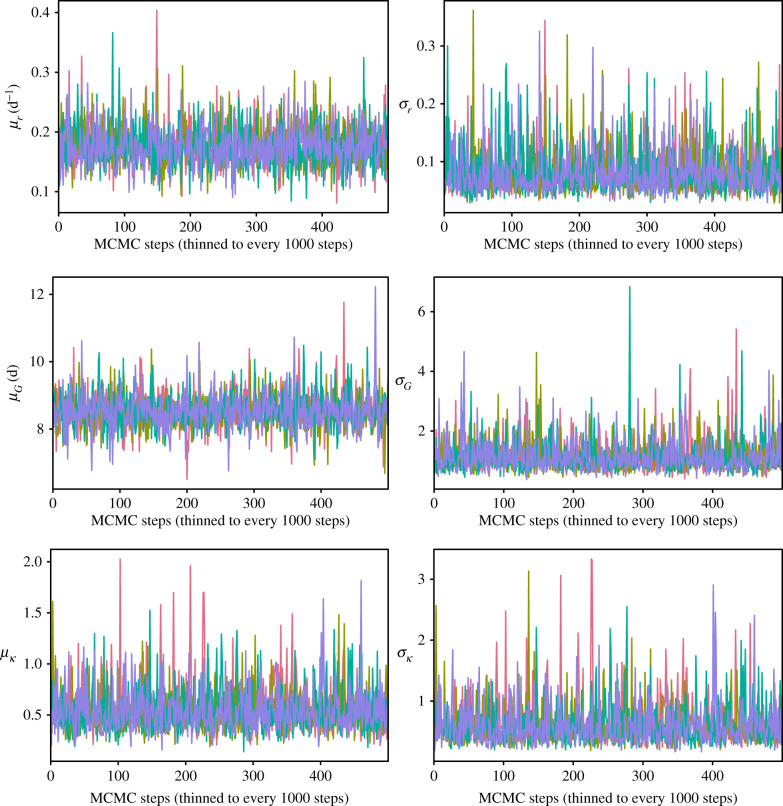
Trace plots of the multilevel model. Each chain is represented by a different colour.

**Figure 5.**
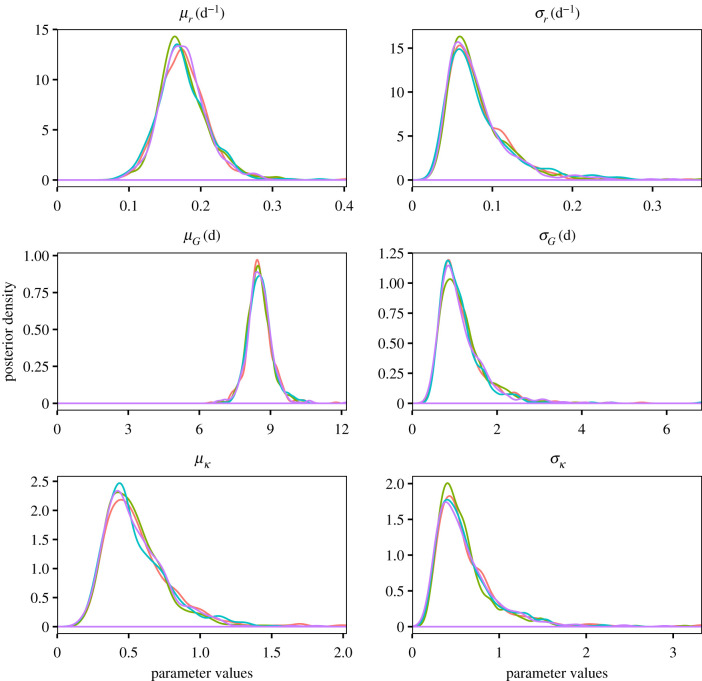
Marginal posterior distributions of the multilevel model. Each chain is represented by a different colour.

## Data accessibility

R code is available in GitHub (https://github.com/parksw3/nCoV_framework).

## Appendix A

See figures 4 and 5.
